# Controlling spike timing and synchrony in oscillatory neurons

**DOI:** 10.1186/1471-2202-12-S1-P223

**Published:** 2011-07-18

**Authors:** Tyler Stigen, Per Danzl, Jeffrey Moehlis, Theoden Netoff

**Affiliations:** 1 Department of Biomedical Engineering, University of Minnesota, Minneapolis, Minnesota 55455, USA; 2 Department of Mechanical Engineering, University of California, Santa Barbara, California, 93106, USA

## 

Many processes in the brain, both normal and pathological, involve oscillations of neuronal populations. The ability to enhance or disrupt neuronal synchrony has been clinically demonstrated to have remarkable effects in a number of neurological disorders including: Parkinson’s disease, Epilepsy, and Depression [1,2]. We have developed an algorithm to control the spike timing of periodically firing neuron using patch clamp and real-time dynamic clamp techniques [3]. Furthermore, this algorithm was expanded to control the relative spike timing between two oscillating neurons. These present the first steps towards more precise population control schemes.

The single cell controller uses the neurons phase response curve and the relationship between the spike advance and the current injection at a given stimulus phase to create a control function [4]. The two cell controller uses the same premise as the single cell controller, but incorporates additional logic to determine the direction and number of periods required to achieve the target phase offset.

We tested our controller using a real-time model neuron and CA1 pyramidal neurons [5]. Noise was added to the model neuron to replicate that seen in biological neurons. In single cell control experiments, the controller could account for ~99% and ~87% of the neurons variance for the model and CA1 pyramidal neuron, respectively. In two cell experiments, we tested the controller using two noisy model neurons and we performed hybrid testing using a model neuron as the leader and a pyramidal neuron as the follower. In the hybrid case, the controller accuracy was moderate, with a normalized vector correlation of 0.69. In the two model neuron case, the controller accuracy was high, with a normalized vector correlation of 0.98 [6]. Using the two neuron model case, we tested the controller for robustness across ISI mismatching and target phase offset. The controller was robust to ISI mismatch as long as it was less than the extremes of the single pulse spike advance, outside that range the accuracy of the controller dropped sharply. The controller accuracy was independent of the desired phase offset between two neurons. In both cases the level of noise injected into the neuron was the primary modulator of controller accuracy.

## Conclusion

Control of neuronal spike timing, even in noisy environments, is possible using the framework presented here. In single cell experiments, approximately 90% of a CA1 pyramidal neuron’s variance could be controlled using this method. The accuracy and robustness of the scheme is limited only by the complexity of the control function, but in practice a simple sigmoid and even linear fits provided excellent control. In two cell experiments, the controller was robust to high levels of noise in both neurons as well as significant mismatching in the natural periods. In principle, the control scheme could be extended to more complicated multi-neuron environments, allowing for control of a population. Empirical evidence suggests that a default parameter set may allow for sub-optimal control across a wide range of neuron types and perhaps allow for stimulation and control of populations using extracellular stimulation.
